# The Importance of Apneic Events in Obstructive Sleep Apnea Associated with Acute Coronary Syndrome

**DOI:** 10.1155/2019/6039147

**Published:** 2019-01-21

**Authors:** George Calcaianu, Didier Bresson, Mihaela Calcaianu, Beatrice Morisset, Tarek El-Nazer, Clara Deodati, Edouard Virot, Daniela Holtea, Carmen Iamandi, Didier Debieuvre

**Affiliations:** ^1^Department of Pulmonary and Sleep Medicine, GHRMSA Mulhouse Hospital, France; ^2^Department of Cardiology, GHRMSA Mulhouse Hospital, France

## Abstract

**Background:**

Obstructive sleep apnea (OSA) is a potential cardiovascular risk factor. However, there is currently no prominent screening strategy for its diagnosis in patients with acute coronary syndrome (ACS). The aim of this study was to establish the impact of apneic events in case of OSA associated with ACS.

**Methods:**

Between January 1st and June 30th, fifty-three subjects with ACS (first acute myocardial infarction) were prospectively evaluated for OSA. Each patient was evaluated by polysomnography (PSG) two months after the ACS.

**Results:**

Mean age of 59±9,6 years, 81,1% males, BMI at 28,5±4,2 kg/m^2^, neck circumference of 42,5±12,6 cm, and waist circumference os 102,5±16,5 cm. The majority of patients (73,6%) had moderate to severe OSA (apnea-hypopnea index (AHI) ≥ 15/h and arousal index ≥ 10/h). We defined the apneic coefficient (AC) as the ratio between apnea index (AI) and AHI. We chose as cut-off the median value of apnea coefficient in our population which was at 37%. The patients with a higher AC (AC ≥ 37% versus AC < 37%) had higher levels of Troponin-I (63,4±63,2 versus 29,7±36,1 ng/mL, p=0,016), higher levels of NT-proBNP (1879,8±2141,8 versus 480±621,3 pg/mL, p=0,001), higher SYNTAX score (15,8±11,5 versus 10,2±5,9, p=0,049), and lower left ventricle ejection fraction (LVEF 53,3±11,4 versus 59,4±6,4%, p=0,023) and were more likely to have a STEMI (21 patients (77,7%) vesus 14 patients (53,8%), p=0,031).

**Conclusion:**

An apneic coefficient (AI/AHI) ≥ 37% is correlated with more severe cardiac impairment, as well as higher hypoxemia and arousal index.

## 1. Introduction

Nowadays, obstructive sleep apnea (OSA) is a common chronic condition, affecting 10% of middle-aged men and 3% of middle-aged women [1].

The clinical guidelines [2, 3] and different other reports highlight that this condition is strongly related to the prevalence and consequences of arrhythmias [4, 5], hypertension [6], stroke [7], and heart failure [8]. The pathophysiological mechanism incriminated in this association is based on endothelial dysfunction [9], coronary plaque burden [10], chronic inflammation, and sympathetic activation [11], secondary to intermittent hypoxemia.

Although the role of OSA as risk factor for cardiovascular disease has been well defined, the description of this sleep-breathing disorder in acute coronary syndrome is less clear and it is based on small population reports [12–15]. Moreover, a recent large study observed that OSA is a predictor of major adverse cardiac and cerebral events in patients who underwent percutaneous coronary intervention (PCI) for ACS, but there is still no consensus on the effectiveness of OSA treatment for patients with ACS [16]. On the contrary, a study of 136 myocardial infarction patients showed lower levels of troponin among OSA patients, suggesting a cardioprotective role of OSA as a “preconditioning factor” [17]. This protective effect of OSA was recently confirmed in patients with ACS, with a 54% reduction in peak cardiac Troponin-I levels in OSA patients than in those without OSA [18].

A recent expert review, based on the last publications, showed a heterogeneity of breathing disturbances associated with OSA and end-organ damage and that therefore a new definition of different phenotypes is needed, based on polysomnographic, clinical, and outcome parameters and not only on AHI [19]. Likewise, the hypopnea thresholds of 3% and 4% seemed to have different cardiovascular consequences [20] and the oxygen desaturation is more pronounced during apnea compared to hypopnea, with longer event duration [21, 22].

Therefore, we hypothesized that a more apneic profile of OSA defined by the ratio between AI and AHI could be better correlated to the severity of cardiac impairment in ACS compared with AHI alone.

## 2. Material and Methods

### 2.1. Study Design

**Figure 1:**
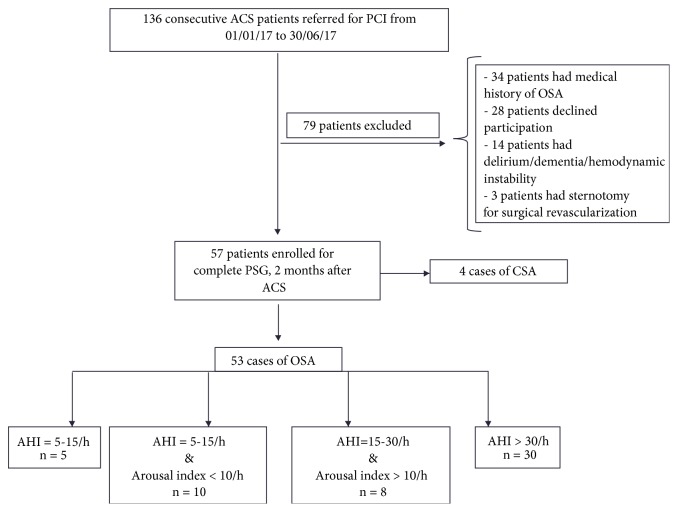
Flowchart: ACS: acute cardiac syndrome; CSA: central sleep apnea; OSA: obstructive sleep apnea; PCI: percutaneous coronary intervention; AHI: apnea-hypopnea index; PSG: polysomnography.

136 consecutives patients with ACS were referred for PCI, between 1st January 2017 and 30th June 2017. Following a screening procedure, 57 patients were enrolled for complete PSG, 2 months after the ACS (Figure 1).

The inclusion criteria included age > 18 years and the presence of first acute myocardial infarction. The exclusion criteria for the current study included the following: previous treatment with CPAP, inability to complete questionnaires, the presence of any previously diagnosed sleep disorder, patients with > 50% central apneas or Cheyne-Stokes respiration, patients with chronic diseases (neoplasms, severe renal insufficiency, and chronic obstructive pulmonary disease), and patients with cardiogenic shock.

All patients underwent systemic sleep apnea screening with polysomnography, performed 2 months after the resolution of the ACS to ensure that fluid accumulation, as a symptom of acute cardiac dysfunction, would not distort OSA diagnosis.

The main objective was the comparison between the apneic coefficient (AC = AI/AHI) and the severity of the coronary disease.

All patients gave written informed consent for the procedures and the research protocol was approved by the Institutional Review Board of the French Learned Society for Respiratory Medicine, Société de Pneumologie de Langue Française (CEPRO 2017-044).

### 2.2. Procedures

The ACS was defined according to the current guidelines [23] and included first ever-acute myocardial infarction (MI) with or without ST elevation infarction and unstable angina. The following data was recorded during hospitalization in the Cardiac Intensive Care Unit:

(i) angiographic data: the Thrombolysis in Myocardial Infarction (TIMI) score to evaluate the angiographic flow (an occluded infraction related artery was defined by a TIMI flow grade 0-1), SYNTAX score, and the infarction related artery (IRA)

(ii) echocardiographic data: LVEF

(iii) laboratory test: Troponin-I level, NT-proBNP, and cholesterol level

(iv) anthropometric data: BMI, neck circumference, and waist circumference.

(v) Sleep validated questionnaires: Epworth Sleepiness Scale (ESS) and Berlin questionnaire.

### 2.3. Polysomnography

Two months after the hospitalization for ACS, all 57 patients underwent a full night PSG in our sleep laboratory, using a Cidelec device (CID102L8D) with monitoring of the electroencephalogram (EEG) using frontal, central, and occipital leads, electrooculogram (EOG), electromyogram (EMG), airflow (by oronasal thermistor and nasal air pressure transducer), thoracic and abdominal respiratory movement (inductance plethysmography), and oximetry. A single EKG lead II was used for cardiac monitoring and the snoring was recorded by a microphone.

Three physicians manually interpreted polysomnographic recordings of 30 sec intervals, in accordance with the AASM 2012 guidelines [24]. Obstructive apnea was defined as the absence of airflow lasting ≥ 10s in presence of abdominal and thoracic movements. Central apnea was defined as the absence of both thoracic and abdominal movements and airflow lasting ≥ 10s. Hypopnea was defined as a reduction in airflow lasting ≥ 10s associated with oxygen desaturation or subcortical arousal. OSA was defined as mild if AHI ≥ 5 and <15/h and moderate to severe if AHI ≥ 15/h and arousal index ≥ 10/h.

We defined the apneic coefficient (AC) as the ratio between AI and AHI. We chose as cut-off the median value of apnea coefficient in our population which was at 37%. Using this cut-off, two groups of patients were formed (AC ≥ 37% versus AC < 37%). All the patients with moderate to severe OSA were proposed to either CPAP therapy or Mandibular Advancement Device (MAD) treatment and we noted the CPAP compliance at 3 months. We defined an optimal adherence to CPAP therapy if the mean duration of nocturnal CPAP therapy was at least 4 hours.

### 2.4. Statistical Analysis

We used SPPS Statistics version 20 for all statistical analysis. Continuous variables are described as mean ± SD or median and interquartile range (IQR) as adequate. Categorical variables were described as numbers and percentages. Correlation coefficients were calculated using Spearman's test. Differences in procedural characteristics between patient groups were analyzed using Mann-Whitney* U* test for continuous data and chi square test for categorical data. A p value < 0,05 was considered significant. All parameters with a p value < 0,20 were entered into the multivariate linear regression analysis.

## 3. Results

### 3.1. Baseline Characteristics

Between January 1st and June 30th, 136 patients with ACS were referred to our Cardiac Intensive Care Unit for primary PCI. 79 patients were excluded according to a priori criteria (34 patients had known history of SAS or ACS, 28 patients declined participation, 14 patients had dementia or hemodynamic instability, and 3 patients had sternotomy or surgical revascularization). Finally, 57 patients were prospectively enrolled to complete the polysomnography study, 2 months after the ACS.

Among them, 39 patients (68,4%) had moderate to severe OSA defined by AHI ≥ 15/h with arousal index ≥ 10/h and only 4 patients presented central sleep apnea (7%). We included only the 53 patients with OSA in our statistical analysis. The baseline demographic, clinical, and procedural characteristics from patients with moderate to severe OSA versus mild OSA patients are listed in Table 1. The mean age was 59 ± 9,6 years, with a male predominance (81%) and BMI at 28,5 ± 4,1 Kg/m^2^. We did not find any statistical difference between the 2 groups (moderate to severe OSA versus mild OSA patients) in terms of severity of ACS.

### 3.2. Results of the Sleep Study

The mean and median value of AHI levels were 35,1/h ± 16,8 and 31/h (range 7 – 77/h), respectively, with 39 patients presenting a moderate to severe OSA (defined by AHI ≥ 15/h and arousal index ≥ 10/h), which represents a prevalence of 73,5% in our population. In terms of screening, we observed that only the Berlin score, completed 2 months after the ACS, was correlated to the severity of OSA (Table 1). We diagnosed only 4 cases of CSA (7% of study population) probably due to the 2 months delay between the ACS and polysomnography.

The excessive daytime sleepiness evaluated by ESS was nonsignificant in both groups of patients (moderate to severe OSA versus mild OSA).

**Figure 2:**
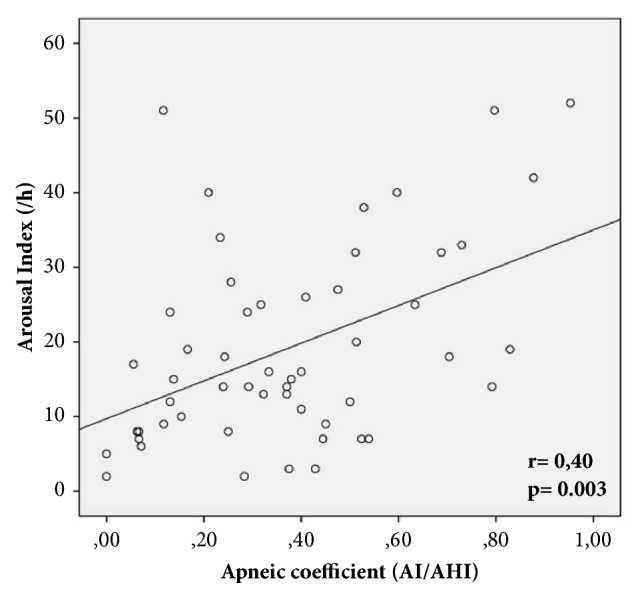
Correlation between arousal index and apneic coefficient.

The apneic coefficient was positively correlated with NT-proBNP (r= 0,31; p=0,33) and arousal index (r=0,40; p=0,003) (Figure 2).

We found a mean and median value of AC levels at 37,3% ± 24,2 and 37% (range 5% – 95%). Using AC levels with a cut-off of 37% we defined two groups: the most apneic patients (AC ≥ 37%) versus the least apneic patients (AC < 37%). We observed that the most apneic patients (AC ≥ 37%) presented significantly worse cardiac impairment with higher frequency of STEMI cases (21 patients (77,7%) versus 14 patients (53,8%), p=0,031), higher SYNTAX score (15,8 ± 11,5 versus 10,2 ± 5,9; p=0,049), higher peak of Troponin-I levels (63,4 ± 63,2 versus 29,7 ± 36,1 ng/mL, p=0,016), higher NT-proBNP levels (1879,9 ± 2141,9 versus 480 ± 621,3 ng/mL, p=0,001), and lower LVEF (53,3 ± 11,4 versus 59,5 ± 6,5%, p=0,023). Moreover, the most apneic patients (AC ≥ 37%) had a significantly higher AHI (40/h ± 14,5 versus 31,3/h ± 16,3; p=0,034) and higher oxygen desaturation index (ODI) (28,8/h ± 15,6 versus 16,4/h ± 13,8; p=0,004) and they were more compliant to CPAP at 3 months with more than 4 hours of CPAP therapy for 19 patients (73%) versus 8 patients (32%); p=0,016 (Table 2). However, all these results are no longer significant after multivariate analysis (Table 3).

## 4. Discussion

OSA is highly prevalent in cardiovascular diseases. Intermittent hypoxia, the hallmark of OSA, causes oxidative stress, inflammation, sympathetic hyperactivity, and endothelial dysfunction which lead to cardiovascular comorbidities [19, 25].

Recent expert review highlighted that the clinical definition of OSA based on the combination of AHI and sleepiness is compromised by the high prevalence of elevated AHI in the general population [19, 26, 27] and the ODI may be a stronger predictor of adverse cardiovascular outcomes than AHI [28, 29]. In this context we hypothesized that a coefficient based on the frequency of pure apneic events could be more reliable to predict cardiac impairment for patients with ACS associated with OSA. Therefore, we defined the apneic coefficient (AC) as the ratio between the AI and AHI. We chose the median value of AC as the cut-off (AC=37%) to evaluate the correlation with the cardiac outcomes after ACS. The polysomnographic evaluation was made 2 months after the ACS to ensure that fluid accumulation, as a symptom of acute cardiac dysfunction, would not distort OSA diagnosis.

We found, after univariate analysis, that the most apneic patients defined by an AC ≥ 37% had a higher level of Troponin-I, higher level of NT-proBNP, higher SYNTAX score, and lower LVEF and were more likely to have a STEMI and a higher ODI. These results are similar to recent studies which showed that OSA is independently associated with major adverse cardiac and cerebrovascular events [30] and could inhibit the recovery of left ventricular function in patients with acute MI [31]. Unfortunately, all these correlations did not remain statistically significant after multivariate analysis probably because of the limited number of patients in our study.

Also, these patients seemed to be more compliant to CPAP therapy. This is probably explained by a significantly more severe sleeping disorder (AHI 40/h ± 14,5 versus 31,3/h ± 16,3 and ODI 28,8/h ± 15,6 versus 16,4/h ± 13,8). Similar to our findings, a recent ancillary analysis of the ISAACC trial showed that protective factors against noncompliance with CPAP treatment were the severity of the disease (high value of AHI and ICU stay length) [32].

We did not find any statistically differences between the groups of mild OSA versus moderate to severe OSA, in terms of coronaropathy markers or cardiac dysfunction after ACS.

A recent study reported that gender in OSA influences the severity of ACS [33]. Our OSA-ACS population presented a large majority of men but without any significant gender-related difference in OSA associated with ACS.

We found a very low level of sleepiness in our population and we did not find any correlation between sleepiness scale evaluated by ESS and the severity of OSA based on AHI. The more complex Berlin questionnaire seemed to better define the more severe OSA patients. Contrary to a recent study [34] we did not find that age or BMI were associated with OSA.

The current study has several strengths including a gold standard polysomnographic evaluation for all 57 patients, performed two months after the ACS to exclude a high prevalence of apneic events due to fluid accumulation secondary to acute cardiac dysfunction. Also, we assessed a broad range of anthropometric, biological, clinical, cardiovascular, and therapeutic variables in our analysis.

Moreover, our study has some limitations. This is a single-center study regarding a small population of patients. Because of a short follow-up period we could not analyze the efficacy and the protective role of CPAP-treatment regarding the cardiovascular status. Repeat coronary angiography was not protocol mandated and therefore we could not examine the follow-up data of the coronary status, regarding the severity of OSA.

In conclusion, in this single-center study we observed that the most apneic patients, defined by an AC ≥ 37%, were associated with a more severe coronary and cardiac dysfunction. The apneic status (AC ≥ 37%) could be a protective factor against the noncompliance to CPAP therapy.

## Figures and Tables

**Table 1:** Baseline characteristics of patients according to severity of OSA. Data are expressed as mean±SD on number (%). ESS, Epworth Sleepiness Scale; PSQI, Pittsburgh Sleep Quality Index; AHI, apnea-hypopnea index; STEMI, ST elevation myocardial infarction; UA, unstable angina; PCI, percutaneous coronary intervention; LVEF, left ventricle ejection fraction.

	Overall (n = 53)	AHI < 15/h and arousal index < 10/h n = 14	AHI ≥ 15/h and arousal index ≥ 10/h n = 39	p-value
Age (yrs)	59,5 ± 9,6	56 ± 9	60,8 ± 9,7	0,102

Male	43 (81%)	11 (78,5%)	32 (82%)	0,528

BMI (kg/m^2^)	28,5 ± 4,1	27 ± 3,9	28,9 ± 4,1	0,203

Neck circumference (cm)	41,5 ± 3,5	40,7 ± 3,7	41,8 ± 3,4	0,343

Waist circumference (cm)	106,4 ± 12,3	99,7 ± 11,1	106,4 ± 11,8	**0,023**

**Laboratory values**				

Creatinine (*µ*mol/L)	79,5 ± 18,8	72,8 ± 12	82 ± 20,3	0,114

NT-proBNP (ng/mL)	1553,7 ± 2830	1263,8 ± 1766,3	1667,9 ± 3169,3	0,845

Peak Troponin I (ng/mL)	44,7 ± 51,6	35,3 ± 35,2	48 ± 56,4	0,755

LDL (g/L)	1,3 ± 0,44	1,3 ± 0,46	1,27 ± 0,44	0,755

HbA1c (%)	6,36 ± 1,3	6,28 ± 1,17	6,39 ± 1,35	0,754

**Sleep related parameters**				

ESS score (baseline)	6,6 ± 3,9	5,7 ± 2,8	6,9 ± 4,2	0,264

High risk Berlin score (baseline)	22 (41,5%)	4 (30,7%)	18 (46,1%)	0,171

PSQI score (baseline)	6,4 ± 3,2	6,25 ± 2,9	6,5 ± 3,3	0,969

ESS score (2 mo. after)	6,1 ± 3,2	4,3 ± 1,4	6,6 ± 3,4	0,054

High risk Berlin score (2 mo. after)	19 (35,8%)	1 (7,2%)	18 (46,1%)	**0,05**

PSQI score (2 mo. after)	6,4 ± 3,2	4,3 ± 2,3	6,8 ± 3,2	0,082

AHI (/h)	35,1 ± 16,8	15,1 ± 3,9	42,3 ± 13,4	**< 0,001**

Arousal index (/h)	22,5 ± 16,5	6,4 ± 4,3	28,2 ± 15,3	**< 0,001**

Mean SpO2 (%)	92,6 ± 1,6	93,4 ± 2,2	92,3 ± 1,2	**0,012**

Time SpO2 < 90% (% sleep time)	10,4 ± 16,5	8,2 ± 22,6	11,2 ± 14	**0,009**

**ACS characteristics **				

SYNTAX score	12,2 ± 9,2	9,8 ± 4,2	13,1 ± 10,4	0,634

Type of ACS				0,776

STEMI	35 (66%)	10 (71,4%)	25 (64,1%)	

NSTEMI	15 (28,3%)	3 (21,4%)	12 (30,7%)	

UA	3 (5,7%)	1 (7,2%)	2 (5,2%)	

Number of diseased vessels				0,94

1	25 (47%)	7 (50%)	18 (46%)	

2	19 (36%)	5 (36%)	14 (36%)	

3	9 (17%)	2 (14%)	7 (18%)

Pre-PCI TIMI flow grade 0-1	23 (43,4%)	4 (28,6%)	19 (48,7%)	0,161

**Echocardiography**				

LVEF (%)	56 ± 9,7	57,5 ± 7,7	55,5 ± 10,4	0,762

**Table 2:** Comparison between the more apneic patients vs. less apneic patients. Data are expressed as mean±SD or number (%). ACS, acute coronary syndrome; STEMI, ST elevation myocardial infarction; UA, unstable angina; PCI, percutaneous coronary intervention; LVEF, left ventricle ejection fraction; ESS, Epworth Sleepiness Scale; AHI, apnea-hypopnea index; CPAP, continuous positive airway pressure.

	Overall (n = 53)	Apneic coef. < 37% n = 26	Apneic coef. ≥ 37% n = 27	p-value
Age (yrs)	59,5 ± 9,6	57,2 ± 9,1	61,8 ± 9,8	0,087

Male	43 (81%)	23 (88,4%)	20 (74%)	0,162

BMI (kg/m^2^)	28,5 ± 4,1	28,1 ± 3,9	28,8 ± 4,4	0,561

Neck circumference (cm)	41,5 ± 3,5	41,2 ± 3,4	41,7 ± 3,6	0,652

Waist circumference (cm)	106,4 ± 12,3	105,7 ± 9,7	107,2 ± 15	0,729

**Laboratory values**				

NT-proBNP (ng/mL)	1553,7 ± 2830	480 ± 621,3	1879,9 ± 2141,9	**0,001**

Peak Troponin I (ng/mL)	44,7 ± 51,6	29,7 ± 36,1	63,4 ± 63,2	**0,016**

**ACS characteristics**				

SYNTAX score	12,2 ± 9,2	10,2 ± 5,9	15,8 ± 11,5	**0,049**

Type of ACS				**0,031 **

STEMI	35 (66%)	14 (53,8%)	21 (77,7%)	

NSTEMI	15 (28,3%)	9 (34,6%)	6 (22,3%)	

UA	3 (5,7%)	3 (11,6%)	0 (0%)	

Number of diseased vessels				0,43

1	25 (47%)	13 (50%)	12 (44,4%)	

2	19 (36%)	10 (38,4%)	9 (33,3%)	

3	9 (17%)	3 (11,6%)	6 (22.3%)	

Pre-PCI TIMI flow grade 0-1	23 (43,4%)	10 (38,4%)	13 (48,1%)	0,332

LVEF (%)	56 ± 9,7	59,5 ± 6,5	53,3 ± 11,4	**0,023**

**Sleep related parameters**				

ESS score (2 mo. after)	6,1 ± 3,2	5,5 ± 2,8	6,7 ± 3,5	0,210

High risk Berlin score (2 mo. after)	19 (35,8%)	6 (33,3%)	13 (52%)	0,183

AHI (/h)	35,1 ± 16,8	31,3 ± 16,3	40 ± 14,5	**0,03**

Arousal index (/h)	22,5 ± 16,5	16,5 ± 11,8	22,3 ± 12,6	0,083

Desaturation index (/h)	22,2 ± 15,8	16,4 ± 13,8	28,8 ± 15,6	**0,004**

Mean SpO2 (%)	92,6 ± 1,6	92,4 ± 1,9	92,7 ± 1,3	0,597

Time SpO2 < 90% (% sleep time)	10,4 ± 16,5	8,7 ± 11,3	12,2 ± 20,7	0,438

Compliance to CPAP treatment (≥ 4 hours of CPAP therapy)	27 (50,9%)	8 (32%)	19 (73%)	**0,016**

**Table 3:** Multivariate analysis of factors associated with AC ≥ 37% versus AC < 37%.

**Variable**	**HR**	**95**%** Confidence Interval **	***p *value**
NT-proBNP	2.88	0.001 – 0,015	0.94

SYNTAX score	0,003	-0,026 – 0,31	0,84

Troponin-I level	0,002	-0,002 – 0,006	0,37

Type of ACS	0,038	-0,440 – 0,364	0,83

LVEF	0,02	-0,044 – 0,003	0,081

Gender	0,332	-0,315 – 0,978	0,97

Age	0,004	-0,045 – 0,053	0,86

CPAP therapy ≥ 4h	0,364	-0,899 – 0,207	0,19

## Data Availability

Data are available on request by contacting the corresponding author, George Calcaianu, at calcaianugeorge@gmail.com.

## References

[1] Peppard P. E., Young T., Barnet J. H., Palta M., Hagen E. W., Hla K. M. (2013). Increased prevalence of sleep-disordered breathing in adults. *American Journal of Epidemiology*.

[2] Somers V. K., White D. P., Amin R. (2008). Sleep apnea and cardiovascular disease: an American Heart Association/american College Of Cardiology Foundation Scientific Statement from the American Heart Association Council for High Blood Pressure Research Professional Education Committee, Council on Clinical Cardiology, Stroke Council, and Council On Cardiovascular Nursing. In collaboration with the National Heart, Lung, and Blood Institute National Center on Sleep Disorders Research (National Institutes of Health). *Circulation*.

[3] Perk J., De Backer G., Gohlke H. (2006). European Guidelines on Cardiovascular Disease Prevention in Clinical Practice (version 2012). The Fifth Joint Task Force of the European Society of Cardiology and other societies on cardiovascular disease prevention in clinical practice (constituted by representatives of nine societies and by invited experts). *Giornale italiano di cardiologia*.

[4] Gami A. S., Pressman G., Caples S. M. (2004). Association of atrial fibrillation and obstructive sleep apnea. *Circulation*.

[5] Kanagala R., Murali N. S., Friedman P. A. (2003). Obstructive sleep apnea and the recurrence of atrial fibrillation. *Circulation*.

[6] Young T., Peppard P., Palta M. (1997). Population-based study of sleep-disordered breathing as a risk factor for hypertension. *JAMA Internal Medicine*.

[7] Bassetti C. L., Milanova M., Gugger M. (2006). Sleep-disordered breathing and acute ischemic stroke: diagnosis, risk factors, treatment, evolution, and long-term clinical outcome. *Stroke*.

[8] Alchanatis M., Tourkohoriti G., Kosmas E. N. (2002). Evidence for left ventricular dysfunction in patients with obstructive sleep apnoea syndrome. *European Respiratory Journal*.

[9] Devulapally K., Pongonis R., Khayat R. (2009). OSA: The new cardiovascular disease: Part II: Overview of cardiovascular diseases associated with obstructive sleep apnea. *Heart Failure Reviews*.

[10] Weinreich G., Wessendorf T. E., Erdmann T. (2013). Association of obstructive sleep apnoea with subclinical coronary atherosclerosis. *Atherosclerosis*.

[11] Bradley T. D., Floras J. S. (2009). Obstructive sleep apnoea and its cardiovascular consequences. *The Lancet*.

[12] Skinner M. A., Choudhury M. S., Homan S. D. R., Cowan J. O., Wilkins G. T., Taylor D. R. (2005). Accuracy of monitoring for sleep-related breathing disorders in the coronary care unit. *CHEST*.

[13] Bahammam A., Al-Mobeireek A., Al-Nozha M., Al-Tahan A., Binsaeed A. (2005). Behaviour and time-course of sleep disordered breathing in patients with acute coronary syndromes. *International Journal of Clinical Practice*.

[14] Moruzzi P., Sarzi-Braga S., Rossi M., Contini M. (1999). Sleep apnoea in ischaemic heart disease: Differences between acute and chronic coronary syndromes. *Heart*.

[15] Mehra R., Principe-Rodriguez K., Kirchner H. L., Strohl K. P. (2006). Sleep apnea in acute coronary syndrome: High prevalence but low impact on 6-month outcome. *Sleep Medicine*.

[16] Lee C.-H., Sethi R., Li R. (2016). Obstructive sleep apnea and cardiovascular events after percutaneous coronary intervention. *Circulation*.

[17] Shah N., Redline S., Yaggi H. K. (2013). Obstructive sleep apnea and acute myocardial infarction severity: Ischemic preconditioning?. *Sleep and Breathing*.

[18] Sánchez-de-la-Torre A., Soler X., Barbé F. (2018). Cardiac Troponin Values in Patients With Acute Coronary Syndrome and Sleep Apnea. *CHEST*.

[19] Randerath W., Bassetti C. L., Bonsignore M. R. (2018). Challenges and perspectives in obstructive sleep apnoea. *European Respiratory Journal*.

[20] Campos-Rodriguez F., Martínez-García M. A., Reyes-Nuñez N., Selma-Ferrer M. J., Punjabi N. M., Farre R. (2016). Impact of different hypopnea definitions on obstructive sleep apnea severity and cardiovascular mortality risk in women and elderly individuals. *Sleep Medicine*.

[21] Kulkas A., Duce B., Leppänen T., Hukins C., Töyräs J. (2017). Severity of desaturation events differs between hypopnea and obstructive apnea events and is modulated by their duration in obstructive sleep apnea. *Sleep and Breathing*.

[22] Campos-Rodriguez F., Gonzalez-Martinez M., Sanchez-Armengol A. (2017). Effect of continuous positive airway pressure on blood pressure and metabolic profile in women with sleep apnoea. *European Respiratory Journal*.

[23] Roffi M., Patrono C., Collet J.-P., Mueller C., Valgimigli M., Andreotti F. (2015). 2015 ESC Guidelines for the management of acute coronary syndromes in patients presenting without persistent ST-segment elevation: Task Force for the Management of Acute Coronary Syndromes in Patients Presenting without Persistent ST-Segment Elevation of the European Society of Cardiology (ESC). *European Heart Journal*.

[24] Berry R. B., Budhiraja R., Gottlieb D. J. (2012). Rules for scoring respiratory events in sleep: update of the 2007 AASM manual for the scoring of sleep and associated events. Deliberations of the sleep apnea definitions task force of the American academy of sleep medicine. *Journal of Clinical Sleep Medicine*.

[25] McNicholas W. T., Bonsigore M. R., Bonsignore M. R., Management Committee of EU COST ACTION B26 (Jan 2007). Sleep apnoea as an independent risk factor for cardiovascular disease: current evidence, basic mechanisms and research priorities. *European Respiratory Journal*.

[26] Kuna S. T., Badr M. S., Kimoff R. J. (2011). An official ATS/AASM/ACCP/ERS workshop report: research priorities in ambulatory management of adults with obstructive sleep apnea. *Proceedings of the American Thoracic Society*.

[27] Heinzer R., Vat S., Marques-Vidal P. (2015). Prevalence of sleep-disordered breathing in the general population: the HypnoLaus study. *The Lancet Respiratory Medicine*.

[28] Seif F., Patel S. R., Walia H. K. (2014). Obstructive sleep apnea and diurnal nondipping hemodynamic indices in patients at increased cardiovascular risk. *Journal of Hypertension*.

[29] Tkacova R., McNicholas W. T., Javorsky M. (2014). Nocturnal intermittent hypoxia predicts prevalent hypertension in the European Sleep Apnoea Database cohort study. *European Respiratory Journal*.

[30] Loo G., Tan A. Y., Koo C.-Y., Tai B.-C., Richards M., Lee C.-H. (2014). Prognostic implication of obstructive sleep apnea diagnosed by post-discharge sleep study in patients presenting with acute coronary syndrome. *Sleep Medicine*.

[31] Nakashima H., Katayama T., Takagi C. (2006). Obstructive sleep apnoea inhibits the recovery of left ventricular function in patients with acute myocardial infarction. *European Heart Journal*.

[32] Florés M., Martinez-Alonso M., Sánchez-de-la-Torre A. (2018). Predictors of long-term adherence to continuous positive airway pressure in patients with obstructive sleep apnoea and acute coronary syndrome. *Journal of Thoracic Disease*.

[33] Sánchez-de-la-Torre A., Abad J., Durán-Cantolla J. (2016). Effect of patient sex on the severity of coronary artery disease in patients with newly diagnosis of obstructive sleep apnoea admitted by an acute coronary syndrome. *PloS One*.

[34] de Batlle J., Turino C., Sánchez-de-la-Torre A. (2017). Predictors of obstructive sleep apnoea in patients admitted for acute coronary syndrome. *European Respiratory Society*.

